# Editorial: Exploring Maternal-Fetal Pharmacology Through PBPK Modeling Approaches

**DOI:** 10.3389/fped.2022.880402

**Published:** 2022-05-18

**Authors:** André Dallmann, John N. van den Anker

**Affiliations:** ^1^Pharmacometrics/Modeling and Simulation, Research and Development, Pharmaceuticals, Bayer AG, Leverkusen, Germany; ^2^Division of Clinical Pharmacology, Children's National Hospital, Washington, DC, United States; ^3^Department of Pediatric Pharmacology and Pharmacometrics, University Children's Hospital Basel, University of Basel, Basel, Switzerland

**Keywords:** PBPK, pregnancy, maternal-fetal, pharmacokinetics, modeling and simulation, physiologically – based pharmacokinetic model

While drug intake during pregnancy is frequent (almost 90%) and still increasing (1, 2), only a small fraction of these drugs (<10%) have been properly studied and labeled for use in pregnant individuals (3). The lack of sufficient information to warrant safe and effective pharmacotherapy during pregnancy constitutes a significant public health challenge. This issue is anything but new. In 1993, the Working Group on Women in Clinical Trials including, amongst others, the commissioner of the Food and Drug Administration (FDA) at that time, Dr. David A. Kessler, stated that “maximizing protection of fetuses from potentially toxic therapies is prudent, and fear of liability is understandable, but the result is that many drugs are ultimately used during pregnancy without reliable data on their maternal and fetal effects” (4). More recently, the response to the COVID-19 pandemic can be seen as another worrisome example illustrating the blatant lack of information to support safe and effective healthcare for pregnant individuals (5–7). While there is some hope that the current paradigm of systematic exclusion will shift toward a fair and responsible inclusion of pregnant individuals in clinical trials (8), other approaches may complement our understanding of maternal-fetal pharmacology and hence improve pharmacotherapy.

Among these approaches, physiologically based pharmacokinetic (PBPK) modeling holds exciting promise (9, 10). PBPK models are compartmental models consisting of a plethora of differential equations describing the pharmacokinetics on a (semi)mechanistic basis, meaning that the relationship between the pharmacokinetics and model parameters is specified in terms of the physical, chemical, and biologic processes that are thought to have given rise to the clinically observed pharmacokinetics. This mechanistic basis brings about a predictive performance superior to that of empirical compartmental models (11–13). PBPK models are increasingly being applied to simulate pharmacokinetics throughout pregnancy (14, 15). This is encouraging in view of the many difficulties in conducting pharmacological studies in pregnant individuals and demonstrates how the potential of PBPK models can be leveraged to refine the knowledge about pre- and perinatal pharmacology, especially when clinical data are sparse, missing, or conflicting.

This article Research Topic aims to promote maternal-fetal PBPK modeling as a high-level tool for gaining a better understanding of drug pharmacokinetics during pregnancy. In the first review, Chaphekar et al. discuss when and how PBPK modeling constitutes an alternative approach to clinical studies and provide a comprehensive summary of the status of human PBPK models for predicting maternal and fetal drug exposure.

Subsequently, several articles of this Research Topic report novel PBPK applications focusing on maternal and/or fetal pharmacokinetics in rats or humans. Personne et al. open this field with a perspective on fetal permethrin exposure throughout gestation in rats. To this end, the authors developed a rat PBPK model for permethrin to estimate placental transfer and prenatal exposure in various tissues including the fetal brain, providing a sound basis for extrapolation to humans.

Another approach to inform placental drug transfer in humans is reported by Mian et al. who present a novel *in silico* cotyledon perfusion model that was used to learn the placental transfer kinetics of acetaminophen from reported data measured in the *ex vivo* cotyledon perfusion system and, upon integration of the learnt transfer kinetics in a whole-body PBPK model, evaluated with clinical data at term delivery.

Along the same line, Liu et al. refined the ordinary differential equation system of an existing pregnancy PBPK model to account for differences in protein binding of drugs between maternal and fetal blood plasma showing that, especially for highly-protein bound drugs, a lower fraction unbound in the fetus vs. mother can markedly affect predicted fetal exposure.

In another work, Amice et al. combined two previously published pregnancy PBPK models for nicotine and cotinine and predict fetal exposure to these substances in plasma and brain after intravenous injection; potential extensions of this model, such as further refinement once more clinical data become available, are also discussed.

While current pregnancy PBPK models typically rely on physiological information from healthy pregnant individuals, they may not fully reflect the underlying physiology of pregnant patients. To tackle this issue, Zhao et al. analyzed serum albumin concentrations collected from large cohorts of pregnant and postpartum women living with HIV and generated functions describing the trajectory of the concentration of each plasma protein in the two cohorts that can be readily utilized for PBPK model development.

This article Research Topic also includes modeling studies with potential implications for clinical practice. Zheng et al. developed a PBPK model for olanzapine; the simulation results in pregnant individuals suggest that dose adjustment can hardly be recommended at the studied stages of pregnancy if treatment before pregnancy was effective and fetal toxicity can be ruled out.

Shenkoya et al. structurally extended a maternal-fetal PBPK model by adding compartments for the lymphatic system and predict the penetration of three antiretroviral drugs in lymphoid tissues-the largest HIV reservoir in the body-indicating that no dose adjustments seem to be necessary in the late third trimester of pregnancy.

Obviously, clinical research involving pregnant individuals can only be carried out within a well-defined regulatory framework. Therefore, two articles from the UK Medicines and Healthcare Products Regulatory Agency (MHRA) and the US Food and Drug Administration (FDA) conclude this Research Topic. Coppola et al. discuss various facets of model evaluation and qualification that are considered necessary if these models are to be used in the context of regulatory application.

Green et al. provide a detailed account of the regulatory framework pertaining to research in mothers, fetuses, and neonates, and discusses multiple aspects of the use of modeling in regulatory submissions concluding that modeling will help fetal pharmacology to quickly move into the mainstream of drug development for the benefit of pregnant individuals and their fetuses.

We hope that this Research Topic will stimulate further research in the field of maternal-fetal PBPK modeling that will ultimately contribute to a more evidence-based approach to pharmacotherapy in pregnancy.

## Author Contributions

AD wrote the first draft of the manuscript. Both authors contributed to the article and approved the submitted version.

## Conflict of Interest

AD is an employee of Bayer AG and uses Open Systems Pharmacology software, tools, and models in his professional role. The remaining author declares that the research was conducted in the absence of any commercial or financial relationships that could be construed as a potential conflict of interest.

## Publisher's Note

All claims expressed in this article are solely those of the authors and do not necessarily represent those of their affiliated organizations, or those of the publisher, the editors and the reviewers. Any product that may be evaluated in this article, or claim that may be made by its manufacturer, is not guaranteed or endorsed by the publisher.
